# Supplementary material for: Pythium banihashemianum sp. nov. and Globisporangium izadpanahii sp. nov.: Two New Oomycete Species from Rice Paddies in Iran
Source: J Fungi (Basel). 2024 Jun 5;10(6):405. doi: 10.3390/jof10060405 (PMC11204656; doi:10.3390/jof10060405)
Supplement: Supplementary file 1 [file jof-10-00405-s001.zip › Supplementary Tables.pdf]

Table S1. List of primers used in this study with their PCR conditions.

Target DNA	Primer name	Primer sequence (5'→3')	Reference	Initial denaturation	Number of cycles	Denaturation	Annealing	Extension	Final extension
ITS[a]	ITS1-o	CGGAGGATCATTACCAC	Bachofer 2004	96 (600)[e]	30	96 (30)	55 (30)	72 (60)	72 (600)
	LR0	GCTTAAGTTCAGCGGGT	Moncalvo *et al.* 1995						
	DC6	GAGGGACTTTTGGGTAATCA	de Cock 1986						
Btub[b]	BT5	GTATCATGTGCACGTACTCGG	Villa *et al.* 2006	96 (600)	30	96 (20)	48 (40 s)	72 (120)	72 (600)
	BT6	CAAGAAAGCCTTACGACGGA							
*cox*1[c]	Oomcox1_Levup	GCT TAA GTT CAG CGG GT	Robideau *et al.* 2011	96 (600)	30	96 (30)	51 (40)	72 (60)	72 (600)
	Oomcox1_Levlo	CYT CHG GRT GWC CRA AAA ACC AAA							
*cox*2[d]	Cox2-F	GGCAAATGGGTTTTCAAGATCC	Hudspeth *et al.* 2000	96 (600)	30	96 (30)	56 (30)	72 (60)	72 (600)
	Cox2-R	CCATGATTAATACCACAAATTTCACTAC							

[a]Internal transcribed spacers 1, 2 and 5.8S gene of rDNA. [b] β-tubulin. [c] cytochrome c oxidase subunit II. [d] cytochrome c oxidase subunit I. [e] Temperature 'ºC' (time s').

Table S2. *Globisporangium* spp. isolate codes and GeneBank accession numbers for phylogenetic analysis comparison.

Species	Isolate Code	GenBank Accession No.			
		ITS	*Btub*	*cox*1	*cox*2
G. abappressorium	CBS 110198	HQ643408.1	KJ595533.1	HQ708455.1	KJ595409.1
G. acanthophoron	CBS 337.29	AY598711.1	KJ595500.1	HQ708460.1	KJ595376.1
G. acrogynum	CBS 549.88	AY598638.1	KJ595458.1	HQ708461.1	AB362324.1
E. anandrum	CBS 285.31	AY598650.1	KJ595450.1	HQ708483.1	AB362328.1
G. attrantheridium	DAOM 230386	HQ643476.1	AB512822.1	HQ708523.1	AB512889.1
G. buismaniae	CBS 288.31	AY598659.1	KJ595493.1	HQ708526.1	KJ595368.1
G. canariense	CBS 112353	HQ643482.1	JX397969.1	HQ708528.1	JX397983.1
G. carolinianum	CBS 122659	HQ643484.1	KJ595551.1	HQ708534.1	KJ595427.1
G. cederbergense	CBS 133716	JQ412768.1	JQ412781.1	JQ412797.1	JQ412805.1
G. cryptoirregulare	CBS 118731	HQ643515.1	GU071888.1	HQ708561.1	GU071763.1
G. cylindrosporum	CBS 218.94	AY598643.1	GU071877.1	HQ708562.1	GU071762.1
G. cystogenes	CBS 675.85	HQ643518.1	KJ595520.1	HQ708564.1	KJ595396.1
G. debaryanum	CBS 752.96	AY598704.1	KJ595523.1	HQ708565.1	KJ595399.1
G. echinulatum	CBS 281.64	AY598639.1	KJ595449.1	HQ708577.1	AB362327.1
G. emineosum	CBS 124057	GQ244427.1	KJ595557.1	GQ244424.1	KJ595432.1
G. erinaceum	CBS 505.80	AY598694.1	KJ595456.1	HQ708578.1	AB362326.1
G. glomeratum	CBS 122644	HQ643542.1	KJ595548.1	HQ708588.1	KJ595424.1
G. heterothallicum	CBS 450.67	AY598654.1	AB512850.1	HQ708596.1	AB512919.1
G. hypogynum	CBS 234.94	AY598693.1	KJ595447.1	HQ708609.1	AB362325.1
G. intermedium	CBS 266.38	AY598647.1	AB512836.1	HQ708616.1	AB507410.1
G. irregulare	CBS 250.28	AY598702.1	GU071886.1	HQ708640.1	GU071760.1
G. iwayamae	CBS 156.64	AY598648.1	JX397965.1	HQ708713.1	JX397979.1
G. kandovanense	CBS 139565	KP723168.1	-	KP938425.1	-
G. kunmingense	CBS 550.88	AY598700.1	KJ595513.1	HQ708716.1	KJ595389.1
G. longisporangium	CBS 122646	HQ643680.1	KJ595550.1	HQ708724.1	KJ595426.1
G. lucens	CBS 113342	HQ643681.1	KJ595540.1	HQ708725.1	KJ595415.1
G. mamillatum	CBS 251.28	AY598703.1	AB512844.1	HQ708731.1	AB512918.1
G. mastophorum	CBS 375.72	AY598661.1	KJ595502.1	HQ708735.1	KJ595378.1
G. megalacanthum	DAOM229154	HQ643693.1	-	HQ708737.1	KJ595435.1
G. middletonii	CBS 528.74	AY598640.1	KJ595457.1	HQ708738.1	AB362318.1
G. minor	CBS 226.88	HQ643696.1	KJ595446.1	HQ708739.1	AB362320.1
G. multisporum	CBS 4570.50	AY598641.1	KJ595455.1	HQ708744.1	AB362319.1
G. nagaii	CBS 779.96	AY598705.1	JX397970.1	HQ708749.1	KJ595402.1
G. nodosum	CBS 102274	HQ643709.1	KJ595531.1	HQ708752.1	KJ595407.1
G. nunn	CBS 808.96	HQ643711.1	-	HQ708755.1	-
G. okanoganense	CBS 315.81	AY598649.1	KJ595498.1	HQ708758.1	KJ595373.1
G. ornacarpum	CBS 112350	HQ643721.1	KJ595535.1	HQ708762.1	KJ595411.1
G. orthogonon	CBS 376.72	AY598710.1	KJ595503.1	HQ708764.1	KJ595379.1

Table S2. Continued.

Species	Isolate Code	GenBank Accession No.			
		ITS	*Btub*	*cox*1	*cox*2
G. oryzicola	UZ382	AB468808.1	-	LC169745.1	AB468930.1
G. paddicum	CBS 698.83	AY598707.1	JX397968.1	HQ708769.1	JX397982.1
G. paroecandrum	CBS 157.64	AY598644.1	GU071882.1	HQ708772.1	DQ071391.1
G. parvum	CBS 225.88	AY598697.1	KJ595445.1	HQ708779.1	AB362322.1
G. perplexum	CBS 674.85	AY598658.1	KJ595519.1	HQ708785.1	KJ595395.1
G. pleroticum	CBS 776.61	AY598642.1	KJ595461.1	HQ708789.1	AB362321.1
G. polare	CBS 118203	AB299390.1	KJ595542.1	-	KJ595417.1
G. polymastum	CBS 811.70	AY598660.1	KJ595527.1	HQ708793.1	KJ595403.1
G. radiosum	CBS 21794	HQ643756.1	MK752967.1	HQ708797.1	KJ595356.1
G. rhizosaccharum	CBS 112356	HQ643760.1	KJ595463.1	HQ708800.1	AB362323.1
G. rostratifingens	CBS 115464	HQ643761.1	KJ595541.1	HQ708803.1	KJ595416.1
G. rostratum	CBS 533.74	AY598696.1	KJ595512.1	HQ708808.1	KJ595388.1
G. segnitium	CBS 112354	HQ643772.1	KJ595537.1	HQ708813.1	KJ595412.1
G. selbyi	CBS 129728	JF836871.1	KJ595471.1	-	JF895532.1
G. sp. *"jasmonicum"*	CBS 101876	HQ643778.1	KJ595530.1	HQ708819.1	KJ595406.1
G. sp. *rooibos2*	STE-U 7550	JQ412777.1	JQ412789.1	JQ412801.1	JQ412813.1
G. spiculum	CBS 122645	HQ643790.1	KJ595549.1	HQ708831.1	KJ595425.1
G. spinosum	CBS 27667	HQ643793.1	KJ595491.1	HQ708833.1	KJ595366.1
G. solare	CBS 119359	EF688275.1	KJ595546.1	-	KJ595421.1
G. splendens	CBS 462.48	AY598655.1	AB512852.1	HQ708836.1	AB512921.1
G. sylvaticum	CBS 453.67	AY598645.1	KJ595507.1	HQ708886.1	KJ595383.1
G. takayamanum	CBS 122491	HQ643854.1	KJ595468.1	HQ708894.1	AB362315.1
G. terrestris	CBS 112352	HQ643857.1	-	HQ708898.1	-
G. ultimum var. *sporangiiferum*	CBS 219.65	AY598656.1	KJ595482.1	HQ708920.1	KJ595357.1
G. ultimum var. *ultimum*	CBS 398.51	AY598657.1	KJ595506.1	HQ708906.1	KJ595382.1
G. uncinulatum	CBS 518.77	AY598712.1	KJ595509.1	HQ708985.1	KJ595385.1
G. viniferum	CBS 119168	HQ643956.1	KJ595544.1	HQ708997.1	KJ595419.1
G. violae	CBS 159.64	AY598706.1	JX397966.1	HQ708999.1	JX397980.1
G. yorkense	C12-118	KY990050.1	KY985299.1	KT692789.1	KY985298.1

Table S3. *Pythium sensu stricto* isolate codes and GenBank accession numbers for phylogenetic analyses comparison.

Species	Isolate Code	GenBank Accession No.			
		ITS	*Btub*	*cox*1	*cox*2
P. acanthicum	CBS 377.34	AY598617.1	KJ595504.1	HQ708458.1	KJ595380.1
P. adhaerens	CBS 520.74	AY598619.1	KJ595510.1	HQ708462.1	KJ595386.1
P. afertile	LEV 2066	HQ643416.1	KJ595563.1	HQ708463.1	KJ595440.1
P. amasculinum	CBS 552.88	AY598671.1	KJ595514.1	HQ708481.1	KJ595390.1
E. anandrum	CBS 285.31	AY598650.1	KJ595450.1	HQ708483.1	AB362328.1
P. angustatum	CBS 522.74	AY598623.1	KJ595511.1	HQ708484.1	KJ595387.1
P. aphanidermatum	CBS 118.80	AY598622.1	KJ595472.1	HQ708486.1	KJ595344.1
P. apiculatum	CBS 120945	HQ643443.1	KJ595547.1	-	KJ595422.1
P. apleroticum	CBS 772.81	AY598631.1	KJ595524.1	HQ708491.1	KJ595400.1
P. aquatile	CBS 215.80	AY598632.1	KJ595481.1	HQ708493.1	KJ595355.1
P. aristosporum	ATCC 11101	AY598627.1	DQ071297.1	HQ708495.1	AB095060.1
P. arrhenomanes	1994-15	AY598628.1	KJ595451.1	HQ708519.1	AF196587.1
P. capillosum	CBS 222.94	AY598635.1	KJ595485.1	HQ708529.1	KJ595360.1
P. catenulatum	CBS 842.68	AY598675.1	KJ595528.1	HQ708536.1	KJ595404.1
P. cf. *dictyosporum*	ADC0114	HQ643495.1	-	HQ708541.1	-
P. chondricola	CBS 203.85	AY598620.1	KJ595480.1	HQ708543.1	KJ595354.1
P. coloratum	CBS 154.64	AY598633.1	KJ595474.1	HQ708553.1	KJ595346.1
P. conidiophorum	CBS 223.88	AY598629.1	KJ595486.1	HQ708559.1	KJ595361.1
P. contiguanum	CBS 221.94	HQ643514.1	KJ595483.1	HQ708560.1	KJ595358.1
P. deliense	CBS 314.33	AY598674.1	KJ595497.1	HQ708566.1	KJ595372.1
P. diclinum	CBS 664.79	AY598690.1	KJ595518.1	HQ708569.1	KJ595394.1
P. dissimile	CBS 155.64	AY598681.1	KJ595475.1	HQ708573.1	KJ595347.1
P. dissotocum	CBS 166.68	AY598634.1	KJ595479.1	HQ708576.1	KJ595351.1
P. flevoense	CBS 234.72	AY598691.1	KJ595488.1	HQ708583.1	KJ595363.1
P. folliculosum	CBS 22094	HQ643540.1	MK752994.1	HQ708584.1	-
P. graminicola	CBS 327.62	AY598625.1	KJ595452.1	HQ708589.1	AF196593.1
P. grandisporangium	CBS 286.79	AY598692.1	KJ595492.1	HQ708590.1	KJ595367.1
P. helicandrum	CBS 393.54	AY598653.1	KJ595453.1	-	AB362329.1
P. hydnosporum	CBS 253.60	AY598672.1	KJ595489.1	HQ708608.1	KJ595364.1
P. inflatum	CBS 16868	AY598626.1	-	HQ708610.1	KJ595352.1
P. insidiosum	CBS 574.85	AY598637.1	KJ595515.1	HQ708613.1	KJ595391.1
P. kashmirense	CBS 122908	HQ643671.1	KJ595553.1	HQ708715.1	KJ595429.1
P. lutarium	CBS 222.88	HQ643682.1	KJ595484.1	HQ708726.1	KJ595359.1
P. lycopersicum	CBS 122909	HQ643683.1	KJ595554.1	HQ708727.1	KJ595343.1
P. macrosporum	CBS 574.80	AY598646.1	AB512842.1	HQ708730.1	AB512916.1
P. marinum	CBS 750.96	AY598689.1	KJ595522.1	-	KJ595398.1
P. marsipium	CBS 773.81	AY598699.1	KJ595525.1	HQ708734.1	KJ595401.1
P. monospermum	CBS 158.73	HQ643697.1	KJ595478.1	HQ708743.1	KJ595350.1

Table S3. Continued.

Species	Isolate Code	GenBank Accession No.			
		ITS	*Btub*	*cox*1	*cox*2
P. myriotylum	CBS 254.70	AY598678.1	KJ595490.1	HQ708748.1	KJ595365.1
P. oligandrum	CBS 382.34	AY598618.1	KJ595505.1	HQ708760.1	KJ595381.1
P. oopapillum	CBS 124053	FJ655174.1	KJ595556.1	FJ655180.1	KJ595431.1
P. ornamentatum	CBS 122665	HQ643722.1	KJ595552.1	HQ708763.1	KJ595428.1
P. pachycaule	CBS 227.88	AY598687.1	KJ595487.1	HQ708767.1	KJ595362.1
P. pectinolyticum	CBS 122643	HQ643739.1	KJ595469.1	HQ708780.1	-
P. periplocum	CBS 289.31	AY598670.1	KJ595494.1	HQ708782.1	KJ595369.1
P. periilum	CBS 16968	AY598683.1	KJ595444.1	HQ708781.1	-
P. phragmitis	CBS 117104	HQ643746.1	EU152854.1	HQ708787.1	AJ890351.1
P. plurisporium	CBS 100530	AY598684.1	KJ595529.1	HQ708790.1	KJ595405.1
P. porphyrae	CBS 369.79	AY598673.1	KJ595501.1	HQ708794.1	KJ595377.1
P. prolatum	CBS 845.68	AY598652.1	KJ595462.1	-	AB362330.1
P. pyrilobum	CBS 158.64	AY598636.1	KJ595477.1	HQ708796.1	KJ595349.1
P. recalcitrans	CBS 122440	DQ357833.1	EF195143.1	-	KJ595423.1
P. rhizo-oryzae	CBS 119169	HQ643757.1	KJ595545.1	HQ708798.1	KJ595420.1
P. salpingophorum	CBS 471.50	AY598630.1	KJ595508.1	HQ708809.1	KJ595384.1
P. schmitthenneri	CBS 129726	JF836869.1	KJ595470.1	-	JF895530.1
P. scleroteichum	CBS 294.37	AY598680.1	KJ595495.1	HQ708812.1	KJ595370.1
P. senticosum	CBS 122490	HQ643773.1	KJ595467.1	-	AB362317.1
P. sp. *spiculacarpum*	CBS 122647	-	-	HQ708815.1	-
P. sp. *tumidum*	CBS 223.94	EF583440.1	-	HQ708816.1	-
P. sukuiense	CBS 110030	HQ643836.1	KJ595532.1	HQ708877.1	KJ595408.1
P. sulcatum	CBS 603.73	AY598682.1	KJ595517.1	HQ708878.1	KJ595393.1
P. tardicrescens	LEV 1534	HQ643855.1	KJ595562.1	HQ708896.1	KJ595439.1
P. torulosum	CBS 316.33	AY598624.1	KJ595499.1	HQ708900.1	KJ595374.1
P. tracheiphilum	CBS 32365	HQ643862.1	-	HQ708903.1	KJ595375.1
P. vanterpoolii	CBS 295.37	AY598685.1	KJ595496.1	HQ708991.1	KJ595371.1
P. volutum	CBS 699.83	AY598686.1	KJ595521.1	HQ709012.1	KJ595397.1
P. zingiberis	CBS 21782	HQ643972.1	-	HQ709013.1	-

Table S4. Base pair differences across *Btub*, ITS, *cox*1, and *cox*2 sequences showing the inter- and intraspecific variation of *Globisporangium izadpanahii* (IZA) and other related species, including *G. coniferarum* (CON), *G. nagaii* (NAG), *G. violae* (VIO), *G. okanoganense* (OKA), *G. canariense* (CAN), *G. monoclinum* (MON), and *G. iwayamae* (IWA).

Region	Alignment length (bp)	Differences IZA/NAG (bp)	Differences IZA/CON (bp)	Differences IZA/VIO (bp)	Differences IZA/OKA (bp)	Differences IZA/IWA (bp)	Differences IZA/CAN (bp)	Differences IZA/MON (bp)	Differences within IZA (bp)
Btub	480	50	12	53	51	56	54	DN	2
ITS	1317	67	52	96	89	99	101	41	2
*cox*1	393	20	8	26	32	26	27	20	0
*cox*2	484	15	8	21	19	30	35	DN*	0
Total	2709	152	80	196	191	211	217	61	0-4

*Data not available

Table S5. Base pair differences across *Btub*, ITS, *cox*1, and *cox*2 sequences showing the inter- and intraspecific variation of *Pythium banihashemianum* (BAN) and other related species, including *P. plurisporium* (PLU), *P. afertile* (AFE) and *P. kashmirense* (KAS).

Region	Alignment length (bp)	Differences BAN/PLU (bp)	Differences BAN/AFE (bp)	Differences BAN/KAS (bp)	Differences within BAN (bp)
Btub	459	7	30	29	0
ITS	877	9	21	21	0
*cox*1	393	6	15	17	0
*cox*2	488	17	16	19	0
Total	2045	39	82	86	0

Table S6. Morphological comparison of the species described in this study with their related species.

Character	*G. izadpanahii*	*G. coniferarum*	*G. nagaii*	*P. banihashemianum* (Group1: G1, Group 2: G2)	*P. plurisporium*	*P. afertile*	*P. kashmirense*
**Cardinal Temperatures (°C)**	5;35;40	15; 30; 40	9; 28; 35	G1:10;35;40<br>G2:5;30;40	15;35; 40	5;25 ;30	5;25;35
**Daily Growth Rate at 25 °C (mm)**	7	6	25	G1:10<br>G2:7	10	18	10
**Colony Pattern on CMA**	Radial	Radial	No data	G1; Radial<br>G2: Uniform	Radial	Compact colony without aerial mycelium	Median
**Hyphae (µm)**	4.3	3.1-7.3	4	G1:3.1<br>G2:3.5	8	6.6	6
**Chlamydospore**		(+), (sub)globose, ovoid	-	-	-	+	-
**Sporangium or Hyphal Swelling Production (µm)**	(+) 13.2	(+) 16.3-17.6	(+)	(+) Variable	(+) Variable	(+) Variable	(+) Variable
**Sporangium or Hyphal Swelling Shape**	Globose	Ellipsoid, globose, ovoid, with pedicel	Ovoid, pyriform	G1: filamentous, inflated;<br>G2: Filamentous, slightly inflated	Filamentous, inflated	Strictly filamentous	Filamentous, slightly inflated

Table S6. Continued.

Character	*G. izadpanahii*	*G. coniferarum*	*G. nagaii*	*P. banihashemianum* **(Group1: G1, Group 2: G2)**	*P. plurisporium*	*P. afertile*	*P. kashmirense*
**Sporangium or Hyphal Swelling Position**	Terminal or intercalary	Mostly terminal	Terminal	-	-	-	-
**Sporangium Proliferation**	-	-	+	-	-	-	-
**Zoospore Production**	-	+	+	+	+	+	+
**Homothallic/Heterothallic**	Homothallic	Homothallic	Homothallic	Homothallic	Homothallic	No sexual structure	Homothallic
**Oogonium Ornamentation**	Smooth with a papillae	Smooth	Smooth	Smooth with 2 papilla	Smooth	No sexual structure	Smooth
**Oogonium Shape and Dimensions (µm)**	Globose, 63	Globose, ovoid, ellipsoid, 25.9-26.7	Globose, sometimes irregular, 14-22	G1: Ovoid, amorphous, 33.3; G2: Globose, 38.3	Globose to obpyriform, 23-35	-	Globose, 16.7
**Antheridium Shape**	Crook-naked, elongated, clavate	Clavate to no specific shape	Clavate	G1 & G2: Clavate, crook-naked	Crook-naked	-	Clavate
**Antheridium Type and Number per Oogonium**	Mostly monoclinous, 1	Monoclinous and diclinous, paragynous, rarely hypogynous, 1-5	Monoclinous, 1 (disappears after fertilization)	G1: Monoclinous and diclinous, 4-8 G2: mostly monoclinous, 1-4	Monoclinous, and Diclinous, 2-12	-	Mostly monoclinous,1-8
**Oospore Type and Dimensions (µm)**	Perfectly plerotic, 63	Aplerotic and plerotic 21.3-23.2	Aplerotic, 12-19	G1: Aplerotic, 32.5 G2: Aplerotic, 32.4	Aplerotic, 26.2-30	-	Aplerotic, 15.4
**Oospore Wall (µm)**	9.2	1.9-3.1	0.8	G1: 1.7 G2:1.5	2.2-3	-	0.7

Table S6. Continued.

**Character**	***G. izadpanahii***	***G. coniferarum***	***G. nagaii***	***P. banihashemianum* (Group1: G1, Group 2: G2)**	***P. plurisporium***	***P. afertile***	***P. kashmirense***
**Number of Oospore per Oogonium**	1	1	1	G1: 1-2<br>G2: 1	1-6	-	1
