# Supplementary figures and images for: Pythium banihashemianum sp. nov. and Globisporangium izadpanahii sp. nov.: Two New Oomycete Species from Rice Paddies in Iran
Source: J Fungi (Basel). 2024 Jun 5;10(6):405. doi: 10.3390/jof10060405 (PMC11204656; doi:10.3390/jof10060405)

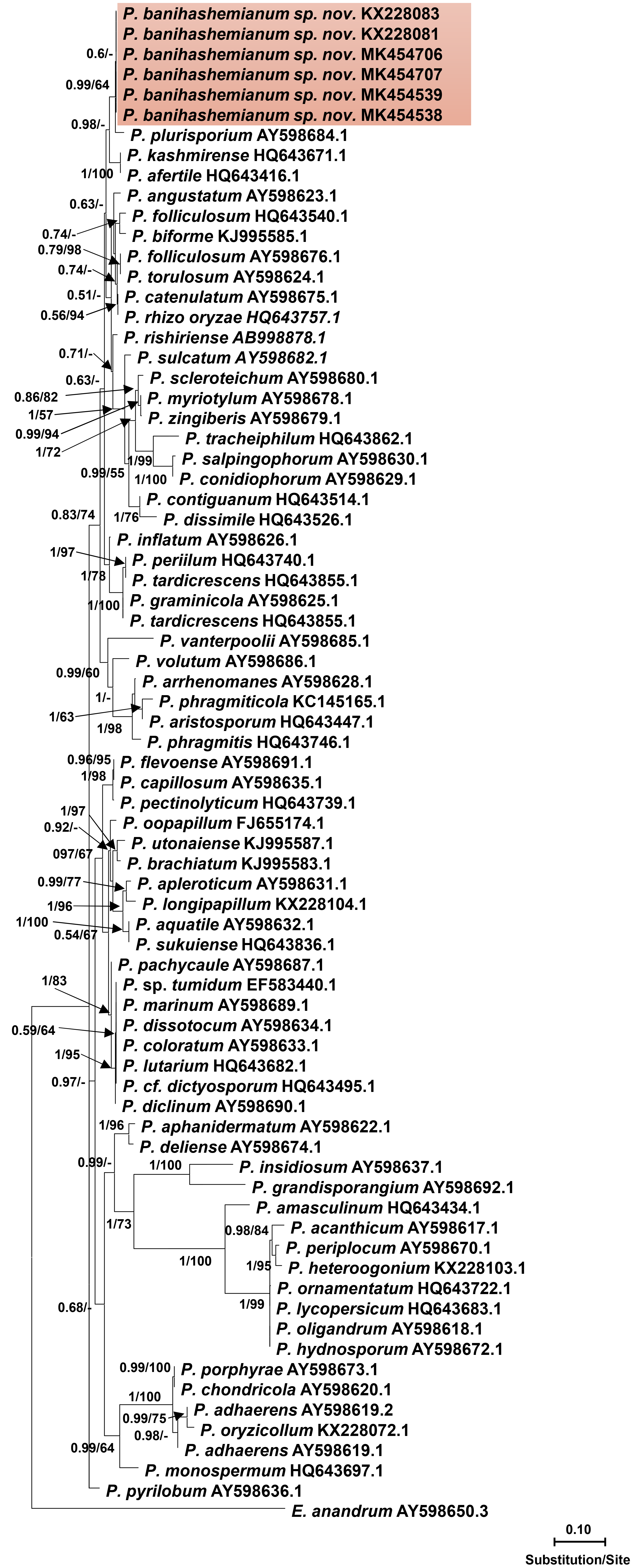

Supplement: Supplementary file 1 [file jof-10-00405-s001.zip › Figure S1.tif]

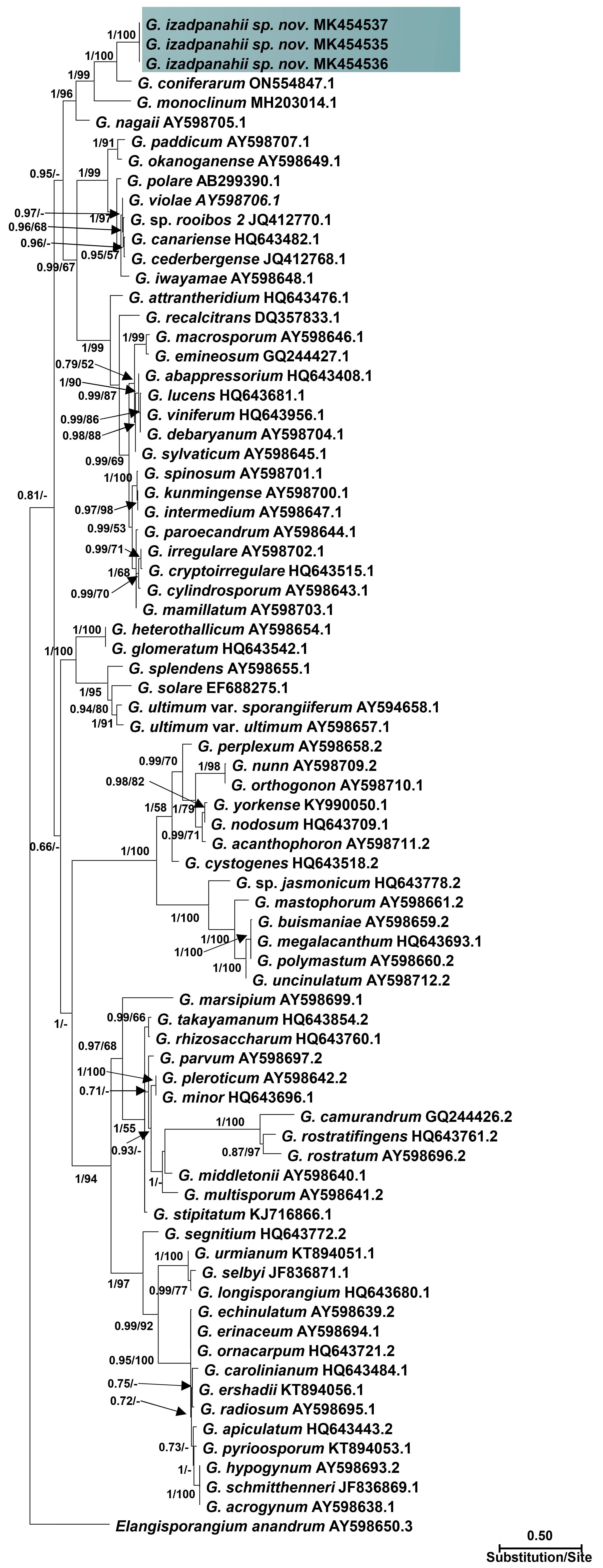

Supplement: Supplementary file 1 [file jof-10-00405-s001.zip › Figure S2.tif]

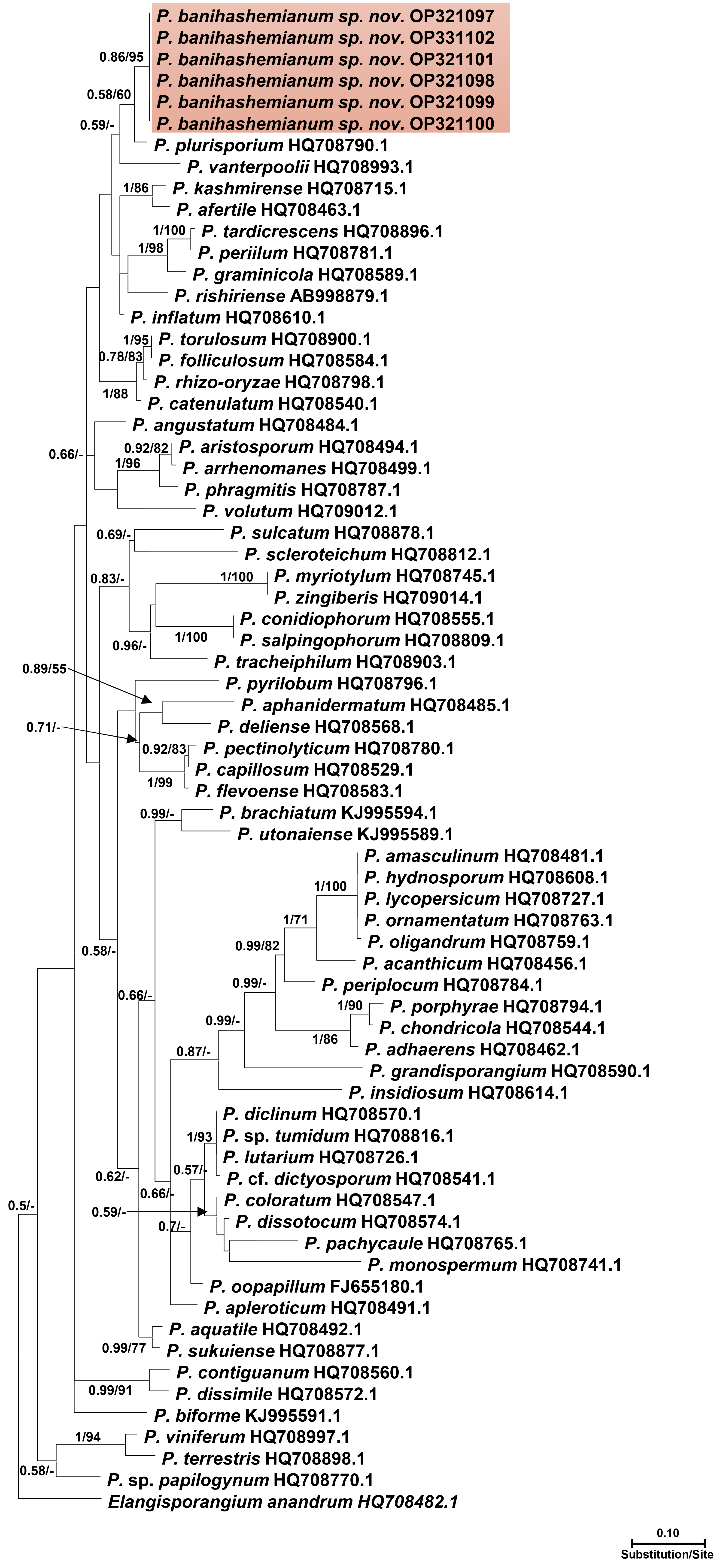

Supplement: Supplementary file 1 [file jof-10-00405-s001.zip › Figure S3.tif]

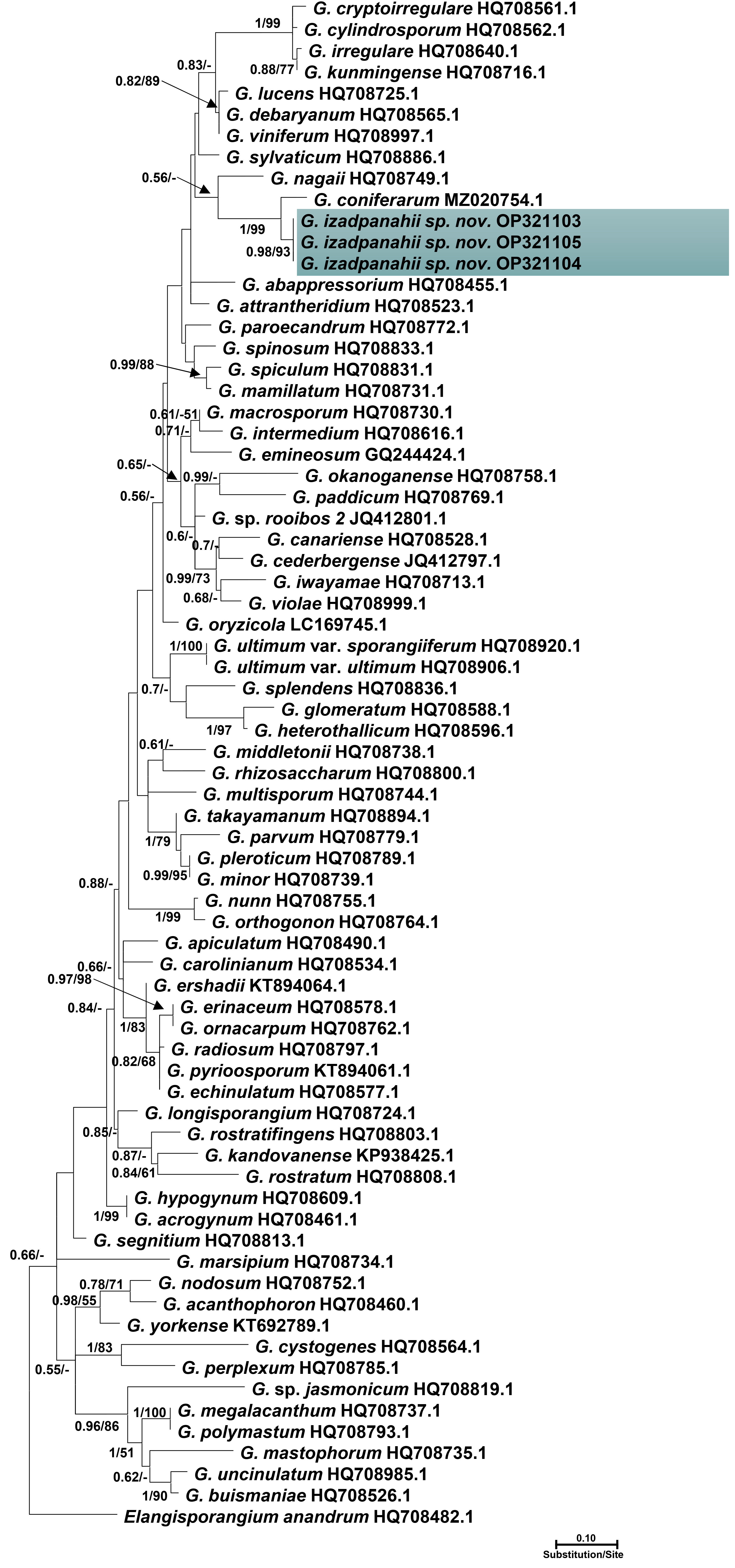

Supplement: Supplementary file 1 [file jof-10-00405-s001.zip › Figure S4.tif]

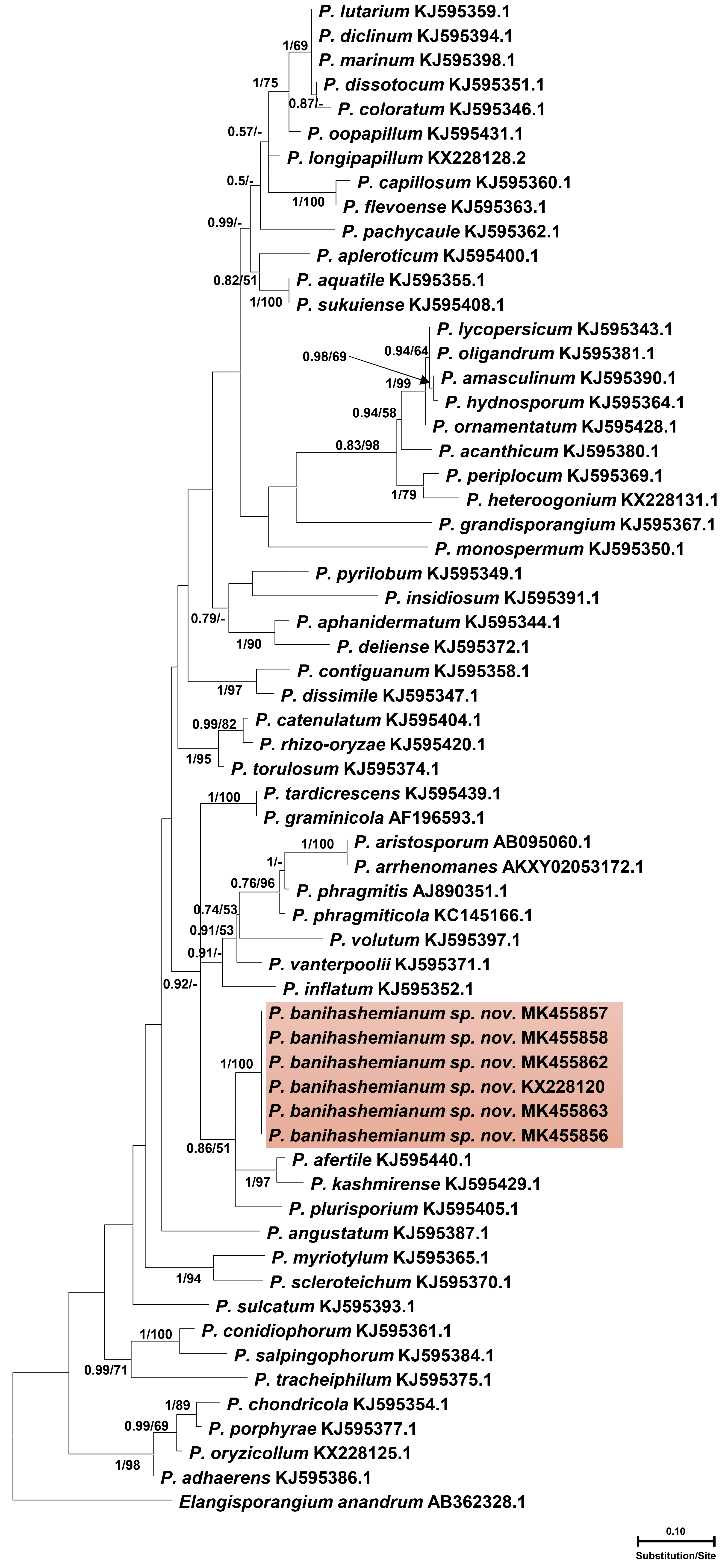

Supplement: Supplementary file 1 [file jof-10-00405-s001.zip › Figure S5.tif]

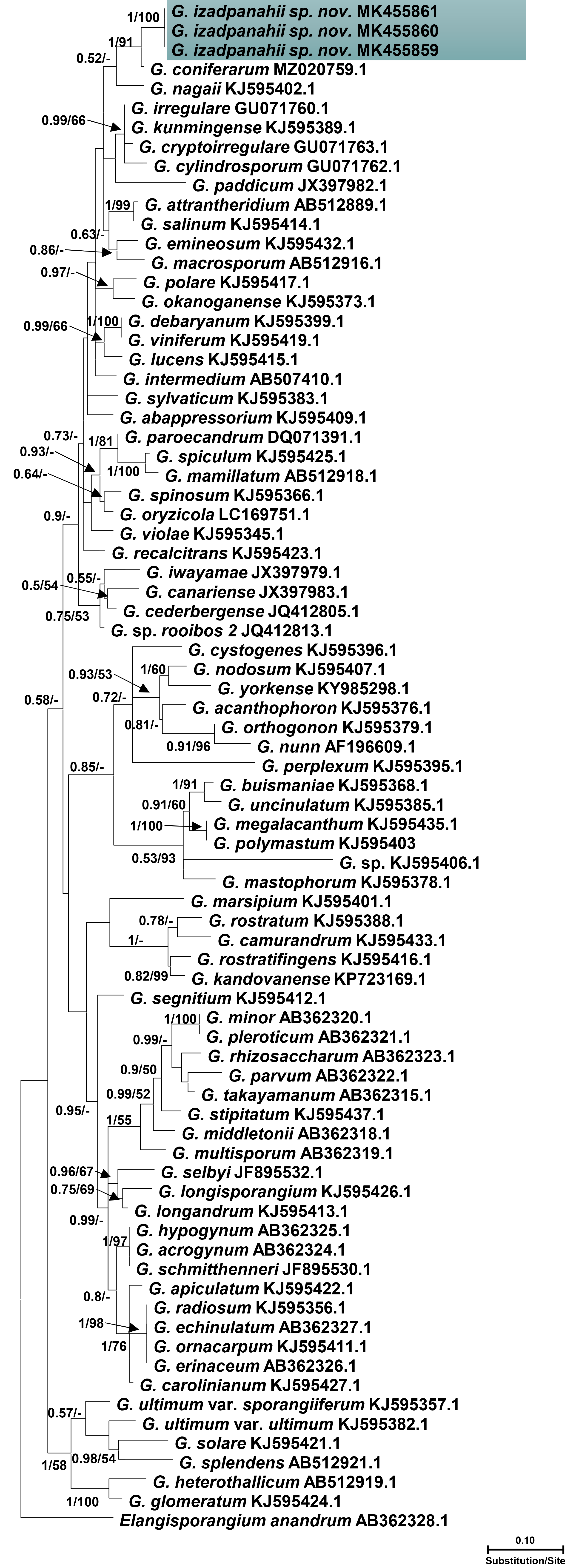

Supplement: Supplementary file 1 [file jof-10-00405-s001.zip › Figure S6.tif]

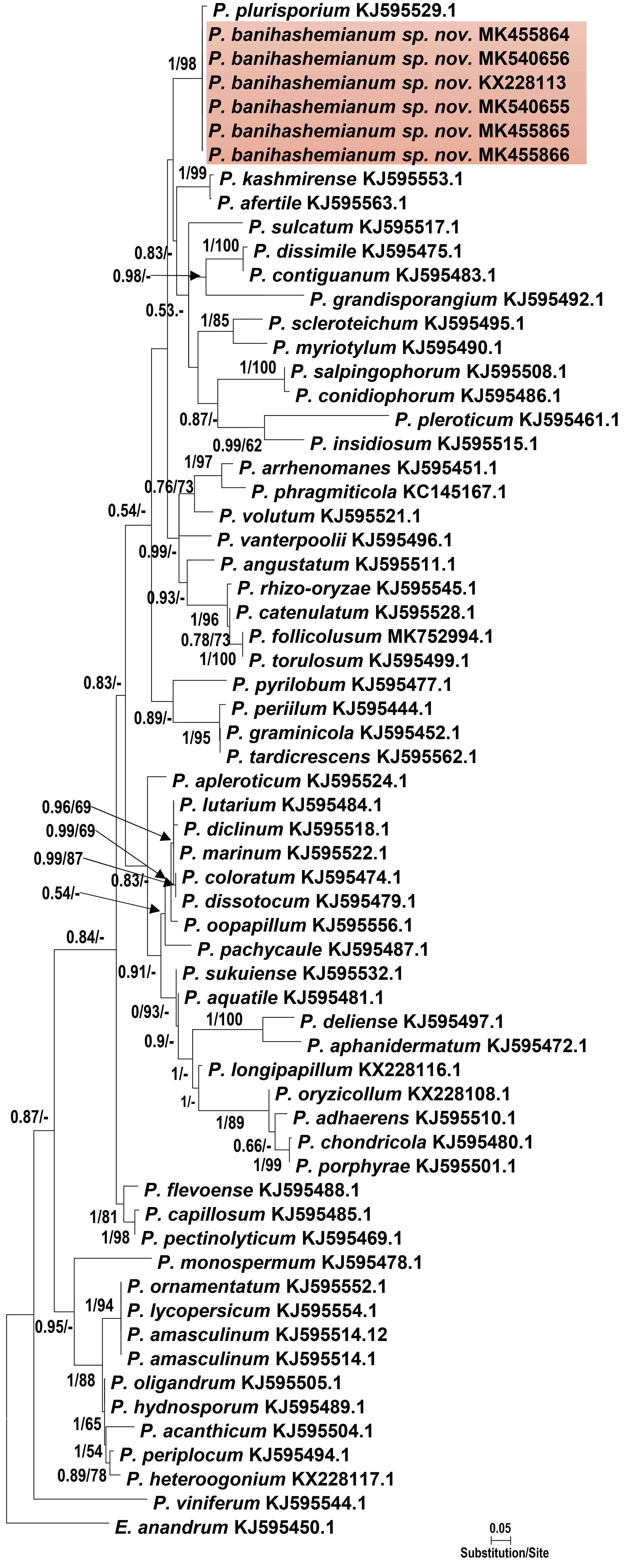

Supplement: Supplementary file 1 [file jof-10-00405-s001.zip › Figure S7.tif]

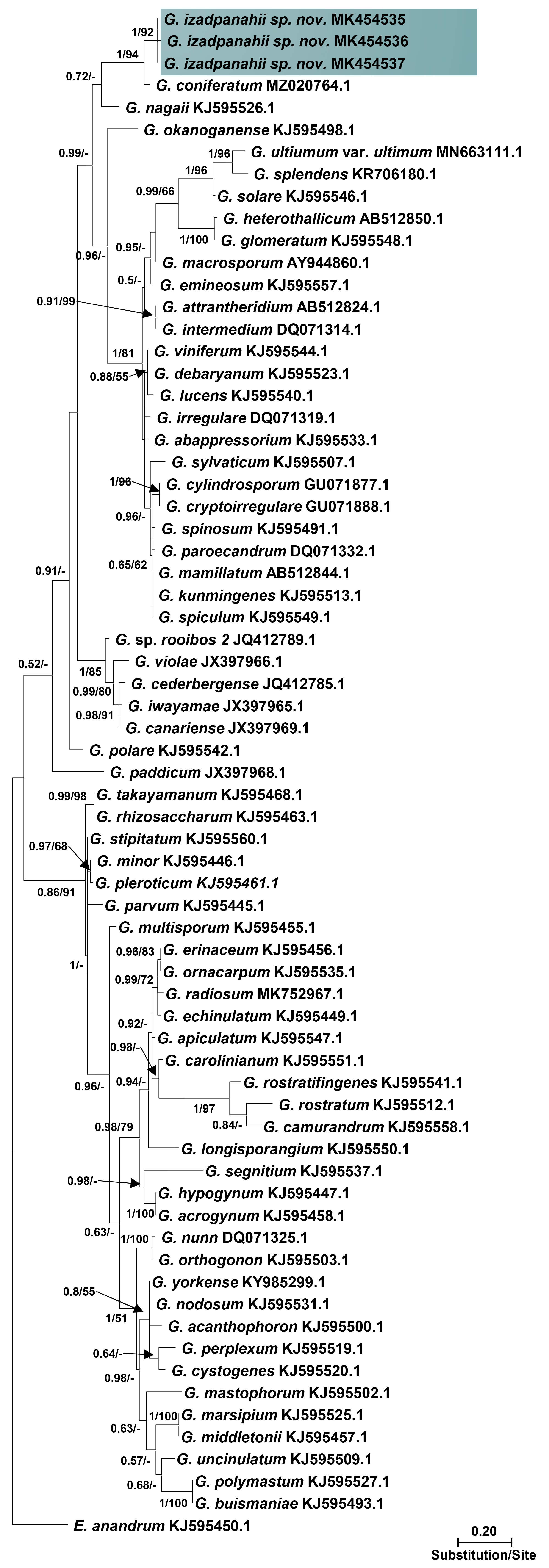

Supplement: Supplementary file 1 [file jof-10-00405-s001.zip › Figure S8.tif]
